# Bacillus of Calmette and Guérin (BCG) and the risk of leprosy in Ciudad del Este, Paraguay, 2016–2017

**DOI:** 10.4178/epih.e2021060

**Published:** 2021-09-08

**Authors:** Nancy Carolina Cuevas, Victor M. Cardenas

**Affiliations:** 1 Alto Paraná District, Ministry of Health, Ciudad del Este, Paraguay; 2 Department of Epidemiology, Fay W. Boozman College of Public Health, University of Arkansas for Medical Sciences, Little Rock, AR, USA

**Keywords:** Leprosy, BCG vaccine, Case-control studies, Epidemiologic studies, Prevention and control, Paraguay

## Abstract

**OBJECTIVES:**

Paraguay has experienced a 35% reduction in the detected incidence of leprosy during the last ten years, as the vaccination coverage against tuberculosis (Bacillus of Calmette and Guérin [BCG] vaccine) reached ≥95% among infants. The objective of this case-control study was to evaluate the protective effect of BCG on the risk of leprosy.

**METHODS:**

We used a population-based case-control study of 20 leprosy confirmed cases reported among residents of Ciudad del Este, Paraguay, diagnosed in 2016–2017. Three controls were selected from a random sample of households from the city. We assessed vaccine effectiveness using 1- odds ratio [OR], and confounding for age, gender, education, occupation, and marital status using stratified and exact logistic regression, and explored if there was effect modification calculating the synergy factor (SF) and relative excess risk due to interaction (RERI).

**RESULTS:**

After controlling for age, gender, education, occupation and marital status, the OR of BCG scar on the risk of leprosy was 0.10 (95% confidence interval [CI], 0.02 to 0.45), for an estimate of vaccine effectiveness of 89.5% reduced risk of leprosy (95% CI, 55.2 to 98.1). There was evidence of heterogeneity by which the effectiveness of BCG seemed stronger among younger persons (Breslow-Day and Z-test of the SF had a p<0.05), and both the RERI and SF indicated a less then multiplicative and additive interaction of BCG and younger age.

**CONCLUSIONS:**

BCG vaccination was associated with a decreased risk of leprosy in the study population, particularly in persons born after 1980.

## INTRODUCTION

A World Health Organization (WHO) Strategic Advisory Group of Experts (SAGE) reviewed in 2017 the evidence of the effectiveness of the Bacillus of Calmette and Guérin (BCG) vaccine, a biologic prepared to prevent tuberculosis infection, in the prevention of leprosy [1]. Based on the evidence of a meta-analysis [2] that included data from 5 randomized trials, 6 cohort studies and 17 case-control studies with a pooled 59% effectiveness (95% confidence interval [CI], 34 to 84), the 2017 WHO SAGE report concluded that “in comparison to the effectiveness of BCG against (tuberculosis), BCG seems to be more protective against leprosy” [1]. As noted in the same report, a BCG trial conducted in Bangladesh reported in 0.4% of contacts receiving the vaccine developed clinical leprosy within 12 weeks of the immunization with BCG [3]. A recent survey conducted by the WHO Global Leprosy Program found that few leprosy-endemic countries have adopted the use of BCG or revaccination of contacts of leprosy cases, including Brazil, Colombia and Peru in the Americas [4].

**Figure 1:**
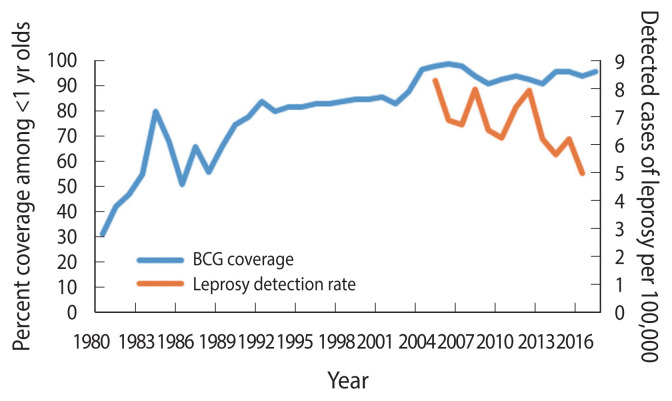
Bacillus of Calmette and Guérin (BCG) vaccination coverage estimates and reported leprosy detection rates, Paraguay, 1980–2017. Source from: World Health Organization. Global health observatory data. Leprosy - number of reported cases by country [5].

The occurrence of leprosy in Paraguay has decreased considerably with detection rates cut by half, between 2005 (8.3 detected cases per 100,000 population) and 2016 (5.0 detected cases per 100,000 population) (Figure 1) [5]. Leprosy occurs at higher rates in states that border Brazil where the detection rates are >10 per 100,000 population [6–8]. In Paraguay, the BCG vaccination started in 1978 [9], targeted newborns at birth and covered all children under 5 years if they had not received the vaccine at birth [10], using vaccines obtained through consolidated purchases of the Pan American Health Organization, or donations from United Nations Children’s Fund, manufactured mostly from the following strains: Pasteur 1173 P2, Statens Serum Institut of Denmark 1331, Glaxo-Evans 1077, and Tokyo 172 [10]. The coverage of BCG vaccination has steadily increased since the 1990s [11]. The 1990 Demographic and Health Survey found a 44% coverage among infants lower than the reported coverage of 75% for the same year [12]. Although the reported figures might overestimate the vaccination coverage, the pattern is consistent with an increasing coverage to nearly universal levels by the 2005 (Figure 1) [5].

Although Brazil bears 12% of the burden of newly detected cases of leprosy in world, in the WHO Region of the Americas, Paraguay comes third behind Brazil and Guyana in terms of detected cases per 100,000 population in 2016 [6]. However, no previous studies on BCG and leprosy were conducted before in Paraguay. We reviewed the scientific literature using an Ovid MEDLINE for the period 1946 and the week ending on June 5, 2021, searching the terms BCG vaccine and leprosy in combination with clinical trials, experimental studies in humans, cohort, case-control, cross-sectional and ecologic or epidemiologic studies and found 111 references, from which 30 were epidemiologic studies, and none of them was conducted in Paraguay. We carried out a case-control study to assess the effectiveness of BCG in Ciudad del Este, Paraguay in 2016–2017.

## MATERIALS AND METHODS

### Study design and population

We conducted an unmatched population-based case-control study using three controls per case in Ciudad del Este (301,815 population), the second largest city in Paraguay. It is located across the border with Brazil and Argentina. The city is close to the Itaipú dam and is free-trade zone that attracts tourists and is the international Friendship Bridge crosses the Paraná River connecting Foz de Iguazú, Brazil and Ciudad del Este, with 70,000 crossings every day.

### Case definition

Cases included were all microscopy confirmed cases of leprosy from January 1, 2016 to July 31, 2017, among residents of Ciudad del Este. All cases were classified according to Ridley and Jopling criteria based on their medical records. All cases were 14 years of age or older.

### Control definition and selection

Controls were residents of Ciudad del Este selected at random as described below, who were at least 14 years of age and who were cognitively able to provide consent. We obtained parental consent for those 14–18 years of age. The controls were selected in a random sample of blocks of Ciudad del Este, using the most recent cartography available at the district office of the Ministry of Health as sampling framework. We selected 30 blocks with probability proportional to size of the blocks, and from each selected city block a starting point was selected at random. Then we visited as many consecutive households walking in eastward direction from the starting point to identify, obtain verbal informed consent and recruit into the study three consenting persons as controls from different households, using the next birthday method. There were no refusals among cases or the potential controls.

### Data collection

One of the authors (NCC) approached all cases and potential controls in June 2017. She invited potential participants to take part in the study and obtained informed written consent. Potential controls were asked if they had ever been diagnosed or told they had leprosy. Both cases and controls were asked if they had received the BCG vaccine and were asked to show their upper arms for presence of a BCG scar.

### Data analysis and sample size

We compared cases and controls for the odds of the presence of BCG scar as well as by age, gender, marital status, last school grade completed, occupation, and contact with a leprosy case, forming dichotomous variables to make the comparisons more stable. We tested the null hypothesis the presence of BCG scar was not associated with the risk of leprosy, i.e., the odds ratio (OR) was 1. We estimated the OR and its 95% confidence interval (CI) and conducted stratified and exact logistic regression analysis that simultaneously controlled for the following potential confounders, age, gender, marital status, education and occupation and examined the presence of heterogeneity using the Breslow and Day test [13]. We estimated the vaccine effectiveness subtracting the OR from 1 [14]. For factors showing a statistically significant heterogeneity, we calculated the relative excess risk for interaction (RERI) in the additive scale [15], and the synergy factor (SF) for the multiplicative scale to measure effect modification [16]. Both measures consider subjects with none of the exposures as referent (OR_00_); the OR for the exposure to the two factors combined (OR_11_) we: (1) subtract (additive scale) the ORs for the presence of one of them in the absence of the other (i.e., OR_10_, OR_01_), or (2) divide OR_11_ by the product of OR_10_ and OR_01_ (multiplicative scale). The 95% CI and the statistical significance of RERI and SF was computed using a method to estimate variance recovery [17] and a standard normal approximation [16], respectively.

Based on preliminary observations of one of the authors (VMC), of a BCG coverage of 80% and assuming the true OR was 0.2 (i.e., BCG effectiveness of 80%), we planned a study with 20 cases and 60 controls. We anticipated such study will be able to reject the null hypothesis that this OR equals 1.0 with probability (power) of 82.6%. The type I error probability α, associated with this test of this null hypothesis was 0.05.

All data analysis were carried out using SAS version 9.4 (SAS Institute Inc., Cary, NC, USA). We calculated 95% CIs around the OR, RERI and SF. Statistical significance was specified as two-sided α=0.05.

### Ethics statement

The study protocol was approved by the Institutional Review Board (IRB) of Faculty for Health Sciences at (Paraguay) National University of the East approved the study (IRB No. # 0401-2019). Informed consent was confirmed by the IRB.

## RESULTS

In 2010–2016 an average of 15 newly detected cases of leprosy have been reported in Ciudad del Este. The detection rate of cases of leprosy in 2016 was 4.9 per 100,000. In 2016, and through June 2017, 20 cases were reported, and all 20 potentially eligible cases and controls agreed to take part in the study. Eleven (55.0%) of the cases were lepromatous leprosy and nine (45.0%) were borderline lepromatous leprosy. Seventy percent were multibacillary. The characteristics of the cases and controls are presented on Table 1. There were no significant differences by age, gender, marital status, schooling, or occupation. However, 2/20 (10.0%) of the cases had a history of contact with a relative known to be a case of leprosy, and such difference was borderline statistically significant (p=0.006), but none of the controls with undefined estimates of the OR and we decided to leave this variable out of the remaining analyses. The most striking difference was on the presence of BCG scar: OR, 0.2; 95% CI, 0.1 to 0.5; p=0.001). The unadjusted vaccine effectiveness was estimated as 1- OR: or 1 - (0.17)=83%; 95% CI, 49% to 94%. The protective effect did not change meaningfully (i.e., >10%) when controlled by age (OR_M-H_=0.2), or gender (OR_M-H_=0.2), education (OR_M-H_=0.2), or occupation (OR_M-H_= 0.2), and only slightly by and marital status (OR_M-H_=0.1). An exact logistic model that simultaneously adjusted for all five covariates, resulted in an estimate of the OR of 0.1 (95% CI, 0.0 to 0.4).

We also found evidence that the effectiveness of BCG varied by age (Table 2). The OR among those less than 40 years of age was 0.0 (95% CI, 0.0 to 0.2), while for those 40 years of age and older was 0.4 (95% CI, 0.1 to 2.0). The p-value of the Breslow-Day for the homogeneity of the OR was 0.03, indicating that the difference by age was statistically significant. Using those without BCG and 40 years of age or older as referent, the OR for the combination of age and BCG shows a reduced risk of leprosy among persons under 40 years of age vaccinated (OR, 0.2), a decreased the risk of leprosy among those with BCG scar and 40+ years (OR, 0.4), an increased risk of leprosy for those younger than 40 without the BCG (OR, 7.0). The SF less than one (i.e., 0.07), implies an effect modification by which the presence of both younger age and BCG resulted in increased protection against leprosy: the actual effectiveness of BCG was 93% in this age group. Conversely, even though 50% of the older controls had a BCG scar, the effectiveness was only 60%. The p-value associated with a normal distribution test showed the positive interaction of BCG with younger age was statistically significant (p<0.05), consistent with the Breslow-Day test, and the 95% CI for the SF did not include the null value (i.e., 1.0) (SF: 95% CI, 0.01 to 0.87). Not surprising, there was also a negative interaction in the additive scale, also called sub-additive as shown by a RERI of −6.2 (95% CI, −42.2 to −0.4). If we were to ignore the effect modification by age, the best estimate of BCG effectiveness controlling for all covariates was 90.0% (i.e., 1.0–0.1; 95% CI, 55.2 to 98.1).

## DISCUSSION

In a population at high-risk of leprosy, located in one of the three countries with the heaviest burden of leprosy in the Americas, we found a decreased risk of leprosy conferred by BCG vaccination. This is the first study of BCG and leprosy in Paraguay, and our findings add to and are consistent with the existing body of knowledge [2]. We found a significant difference in the protection provided by age, by which BCG among younger individuals resulted in a reduced risk, but not among older individuals. The findings of heterogeneity of the effectiveness of the BCG on the risk of leprosy could be interpreted as the protective effect wanes with time since vaccination and are consistent with the results of other epidemiologic studies, including a large case-control study conducted in Brazil, which found the protection declined from 86% to 54% and further to only 32% in 18–29 years, 30–39 years and 40+ year-olds [18]; another case-control study in India, which found the BCG vaccine was more effective in those under 20 years compared with those ≥20 years of age (effectiveness of 61% and 43%, respectively) [19], and a randomized trial in Malawi that found a 60% effectiveness among children under 15 years of age, and only 27% among those ≥15 years of age [20]. It has been observed that booster BCG vaccination increased the effectiveness against leprosy [21–24]. Since there has not been any revaccination policy in Paraguay during adulthood like in Brazil, in particular targeting household contacts, the difference in effectiveness observed by age could be a proxy of differences in time since vaccination. Most of the cases in our case series were multibacillary, and previous studies have reported higher effectiveness of BCG against this form of leprosy [19,25].

Our estimate of effectiveness is similar to those of most epidemiologic studies, including field trials [20,22,23,26–31], cohort studies [23,29,32–35], case-control studies [18,19,36–48], most of them summarized in a meta-analysis [2].

### Limitations

We did not include participants under 14 years of age in our study, as there were no cases under that age, which limits the generalizability to incidence in older children and adults in Paraguay. Although known cases of leprosy under 14 years of age are rare, they could also be overlooked. The leprosy program could be under diagnosing paucibacillary leprosy; however, it may happen in both Paraguay and Brazil, as the proportion of paucibacillary cases was similar in Foz de Iguazú [9]. Our study was small and was not planned to fully assess the effect modification of the age of the participants of the effectiveness of the BCG vaccination. It is unlikely that there has been any shift in quality of BCG since the vaccines strains of four laboratories have accounted for about 90% of BCG supplies over time, and no differences in effectiveness have been found across them. The differences are unlikely explained just by chance, but future larger studies, specifically designed to assess the interaction of age and BCG in the multiplicative scale, requiring about 320 cases and 960 controls, if using a 1:3 ratio to maximize the statistical power, assuming the same age distribution, and coverage of BCG (70%) as found in the study population, are needed [49]. Further studies are needed in Paraguay, possibly assessing use of BCG and rifampin among contacts.

In conclusion, this small study confirms the potential impact of BCG vaccination to prevent leprosy. We recommend exploring the adoption of strategies in place in Brazil that complements multidrug therapy with contact tracing and BCG targeted booster vaccination.

## Figures and Tables

**Table 1:** Comparison of leprosy cases and controls by demographics, contact with other leprosy cases, and BCG scarring, Ciudad del Este, Paraguay, 2017

Characteristics	Case (n=20)	Control (n=60)	OR (95% CI)	p-value
Age (mean), yr	44.6	41.8	-	0.60
14–39	10 (50.0)	30 (50.0)	1.0 (reference)	
40–79	10 (50.0)	30 (50.0)	1.0 (0.4, 2.7)	>0.99

Gender				
Men	13 (65.0)	28 (46.7)	2.1 (0.7, 6.1)	0.20
Women	7 (35.0)	32 (53.3)	1.0 (reference)	

Marital status				
Married/living with a partner	13 (65.0)	30 (50.0)	1.9 (0.7, 5.3)	0.30
Single/widowed/divorced	7 (35.0)	30 (50.0)	1.0 (reference)	

Education				
≤6th grade	10 (50.0)	25 (41.7)	1.4 (0.5, 3.9)	0.60
>6th grade	10 (50.0)	35 (58.3)	1.0 (reference)	

Occupation				
Unemployed/street vendor	2 (10.0)	3 (5.0)	2.1 (0.3, 13.6)	0.60
Other	18 (90.0)	57 (95.0)	1.0 (reference)	

Living with someone with leprosy				
Yes	2 (10.0)	0 (0.0)	∞ (0.9, ∞)	0.006
No	18 (90.0)	60 (100)	1.0 (reference)	

BCG scar				
No	14 (70.0)	17 (28.3)	1.0 (reference)	0.001
Yes	6 (30.0)	43 (71.7)	0.2 (0.1, 0.5)	

Values are presented as number (%).

BCG, Bacillus of Calmette and Guérin.

**Table 2:** Effect modification between age and BCG scarring on the risk of leprosy, Ciudad del Este, Paraguay, 2016–2017

BCG scar	Age (yr)				OR (95% CI) for age within stratum of BCG^1^

	≥40		<40		
	
	Case/Control	OR (95% CI)	Case/Control	OR (95% CI)	
Absent	7/15	1.0 (reference)	7/2	7.0 (1.2, 59.9)	7.0 (1.2, 59.9)
p-value				0.03	0.03

Present	3/15	0.4 (0.1, 2.1)	3/28	0.2 (0.0, 1.0)	0.5 (1.0, 3.5)
p-value		0.30		0.06	0.50

BCG, Bacillus of Calmette and Guérin; OR, odds ratio; CI, confidence interval.

1 Relative excess risk due to interaction=0.2–0.4–7.0+1.0=−6.2 (95% CI, −42.2 to −0.4); There is a negative interaction in the additive scale, also called sub-additive; Measure of interaction on the multiplicative scale= (0.2/0.4×7.0)=0.07 (95% CI, 0.10 to 0.87); There is a negative interaction in the multiplicative scale; The attributable proportion due to the interaction was −24.3 (95% CI, −100.0 to −3.2).
